# Effectiveness of “Be SAFE Drowning Prevention and Water Safety Booklet” Intervention for Parents and Guardians

**DOI:** 10.18502/ijph.v49i10.4695

**Published:** 2020-10

**Authors:** Noor Hamzani FARIZAN, Rosnah SUTAN, Kulanthayan KC MANI

**Affiliations:** 1. Department of Community Health, Faculty of Medicine, University Kebangsaan Malaysia, Kuala Lumpur, Malaysia; 2. Department of Community Health, Faculty of Medicine and Health Sciences, University Putra Malaysia, Serdang, Malaysia

**Keywords:** Health education, Drowning, Prevention, Knowledge, Attitude, Practice

## Abstract

**Background:**

We aimed to assess the effectiveness of the health educational booklet intervention in improving parents/guardian’s knowledge on prevention of child drowning and, the perception of drowning risk and water safety practice.

**Methods:**

A quasi-experimental study was conducted in year 2017 in Selangor, Malaysia among 719 parents/guardians of primary school children. The parent/guardians were randomly assigned as the intervention groups and were given a health educational Be-SAFE booklet on drowning prevention and water safety. The pretest was conducted before the intervention and posttest was done one month of intervention. The data collection tool was using a validated questionnaire on knowledge, attitude and practice for drowning prevention and water safety.

**Results:**

There were 719 respondents (response rate of 89.9%) participated at baseline and 53.7% at end line (after the intervention). Significant differences found in knowledge, attitudes and practice on drowning prevention and water safety for the intervention and control groups after the intervention (*P*<0.001). There was a significant difference in mean scores for knowledge and attitude before and after the intervention, whereas no significant findings noted for practices (*P*<0.001).

**Conclusion:**

Be SAFE booklet contributed to the increase in parents/guardian’s knowledge and attitudes towards drowning prevention and water safety to prevent the risk of child drowning.

## Introduction

Globally, drowning was demanded as a significant health problem and amongst the top ten leading causes of death among children and young people in all regions. Drowning was the major cause of death from unintentional injury among children aged less than 14 yr with an estimated 129,553 deaths worldwide in the year 2012 (1).

Drowning can happen in many different ways, hence it needs a range of prevention strategies to target the biggest risks especially with related to children (2). Educational intervention found to be cost-effective in the prevention of child injury, and community-based intervention has a good uptake and possibility of improving parental supervision practices and reducing childhood drowning (2–4). Education campaigns and programs to individual and community on awareness of drowning, the risk associated with drowning and learning water survival skills are promising strategies to reduce drowning (4–7). Thus, preventive measures such as health education drowning prevention guideline can be established and it may minimize or reduce the problem of drowning among children. Childhood drowning in Malaysia still does not receive ample attention (8). Very minimal studies were conducted in relation to drowning in this country and a major ignorance of the reality on this issue might due to very limited data available on drowning incidents, lack of information on the mechanism of the drowning events and little is known on people perceptions related to drowning risk.

Hence, we aimed to examine drowning risk knowledge, attitude and practice of water safety among the parents/guardians of primary school children in Malaysia and assess the effectiveness of drowning prevention and water safety intervention educational package.

## Materials and Methods

### Study design

This was a quasi-experimental intervention study involving pre and post-test carried out among parents/guardians of primary school children.

### Study population

Hulu Langat sub-district of Selangor has 86 public primary school and is the 2nd sub-district with the highest number of primary schools in Selangor.

Simple random sampling was conducted, and school were identified and selected based on the preference to involve in the study. From multistage random sampling, school children were randomly chosen from a selected school as the proxy for their parents and guardians to be invited to participate in this study. The sample met the following inclusion criteria: Parents/guardians with at least one child study in selected school, and willing to participate in the study by signing the consent form.

### Estimation of sample size

The proportions method was used to determine the sample size needed to estimate the prevalence of knowledge and practice in drowning prevention among parents/guardians of primary school children (9). Where *n* is the sample size required, *z* is the value of standard normal distribution corresponding to 95% confidence interval, *P* is the estimated proportion knowledge and practice in drowning prevention among parents/guardians of primary school children, q is 1-p, and d is the margin of error allowed (0.05). Using these values, the required sample size is 385 or 400. The actual sample size was calculated as 400 and were times with study effect it became as the required sample was 800.

### Respondents and recruitment

**Fig.1:**
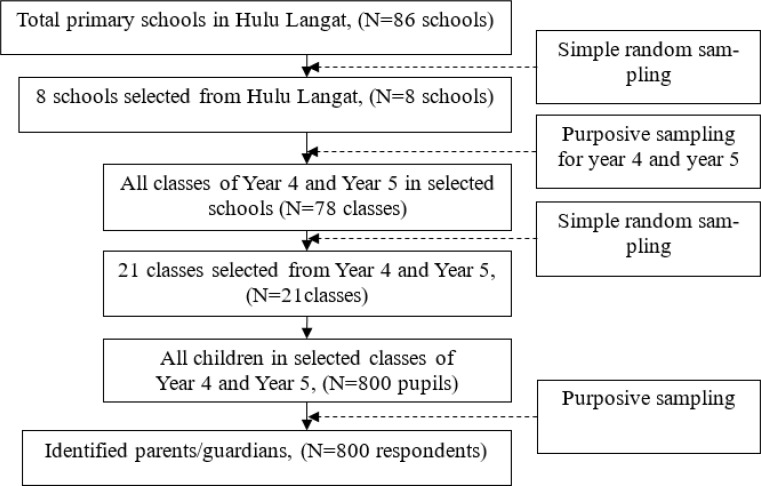
Identification of participants

Researcher identified parents/guardians from randomly selected primary school and they were invited to participate in the study. Permission to involve school as data collection point and students to reach their parents/guardians were granted by Ministry of Education Malaysia (MOE). Cooperation and collaboration with school teachers also were obtained to deliver a message to parents/guardians on conducting the study. The questionnaire for parents/guardians were delivered by their children and returned back either directly to the researcher or to the class teacher. The schematic diagram on the identification of parents/guardians of primary school children involving multi-stage random sampling were shown in Fig.1. Multi-stage random sampling was conducted in stages using smaller and smaller sampling units at each stage as from schools to classes, to children and finally to the identification of parents/guardians.

There were 800 parents/guardians identified to be eligible to participate in this study, 81 refused to take part in the reason that they were not interested. Finally, 719 parents/guardians were agreed to participate and completed the assessment.

### Study instruments and measures

The respondents (N=800) were allocated according to the control group (n=400) and intervention groups (n=400); namely booklet group (n=200) and booklet & seminar group (n=200). Parents/guardians allocated in the control group did not receive any health educational booklet or program during the study periods. Participants in booklet group received health education Be SAFE booklet, and booklet & seminar group received the same health education (Be SAFE booklet) during the Be SAFE Seminar.

The Be SAFE seminar was an enhancement to the booklet intervention and was devoted to the presentation of the main section of the booklet by which lasted for about 30 – 45 min. At the end of each presentation, there was a practical demonstration of drowning prevention and water safety. All intervention participants received a brief explanation on the use of the Be SAFE booklet. The booklet was in 20 pages-color, illustrated with a proper image and was written in the national language, Malay Language (Bahasa Melayu). Self-administrated questionnaire on knowledge, attitude and practice on drowning prevention and water safety were used as a survey tool for parents/guardians and the developed Be SAFE booklet were used as instruments of intervention. At baseline, all three groups answered a questionnaire on KAP of drowning prevention and water safety (pre-test) and a month after the intervention, respondents completed the same questionnaire for a post-test assessment.

### Ethical approval

The study was approved by Research Ethics Committee, The National University of Malaysia (UKM PPI/111/8/JEP-2016-594).

## Results

### Response rate

**Fig. 2:**
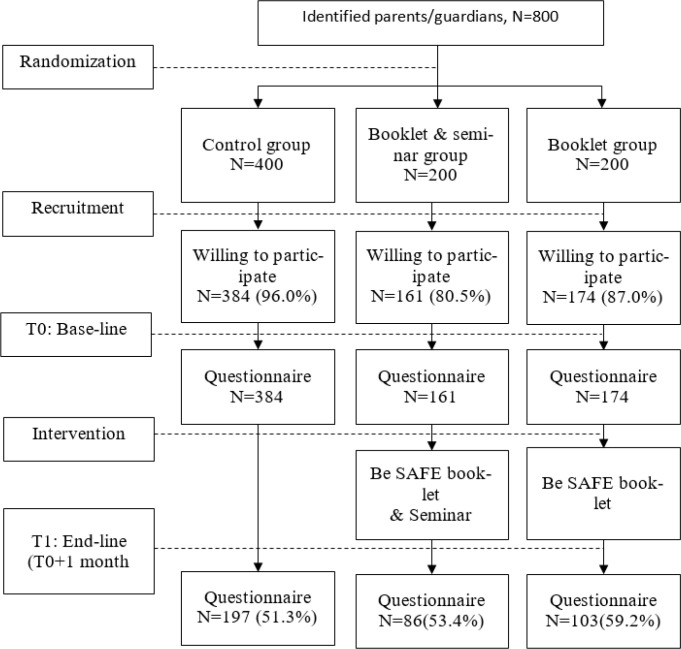
Response rate by groups involved in the study

There were 800 eligible parents/guardians to participate in this study and 719 (89.9% total response rate) completed the baseline assessment (384 in control group, 174 in booklet group and 161 in booklet & seminar group. Out of 719 participants, 386 completed end-line assessment (a month after intervention) with 53.7% total response rate. The details of response rate for each group were showed in Fig. 2.

### Socio-demographic characteristics of parents/guardians

Majority of the respondents were Malay and a mother of primary school children. The age of respondents ranged from 21 to 56 yr with mean 41.42 (±6.097). Table 1 shows the summary of the respondent’s demographic characteristic.

**Table 1:** Characteristics of parents/guardians enrolled in the study

***Variable***	***N***	***%***	***Mean***	***SD***
Age (yr)			41.42	6.097
Ethnicity				
Malay	680	94.6		
Chinese	5	0.7		
Indian	11	1.5		
Others	23	3.2		
Number of children			1.13	0.582
Relationship with school children				
Father	308	42.8		
Mother	390	54.2		
Guardian	19	2.7		
Other	2	0.3		
Educational level				
No schooling	5	0.7		
Primary school	27	3.8		
Secondary school	309	42.9		
Certificate/Diploma	177	24.6		
Degree	160	22.3		
Master and above	41	5.7		
Occupation				
Not working	20	2.8		
Housewife	144	20.1		
Self-employed	88	12.2		
Government sector	198	27.5		
Private sector	257	35.7		
Others	12	1.7		
Household income				
Less than RM7 000	560	77.9		
RM7 000 - 15 000	135	18.8		
More than RM15 000	24	3.3		

### The outcome of the intervention Comparison of knowledge, attitude and practice between groups

One way between-group ANOVA tests were conducted whether there are significant differences in the mean knowledge, attitude and practice scores across three study groups. There were statistically significant differences found in knowledge F (2, 386) = 120.26, *P*<0.001, attitudes F (2, 386) = 19.14, *P*<0.001 and practice F (2, 386) =11.67, *P*<0.001 on drowning prevention and water safety for the three study groups after the four weeks intervention.

### Comparison of knowledge, attitude and practice within groups

Change in the knowledge, attitude and practice of the parents/guardians from baseline to four weeks after intervention for the control and intervention groups were presented respectively in Table 2. The eta squared was 0.11 indicated the small effect size. The result indicated that there was a large effect, with a substantial difference in the knowledge scores obtained before and after the intervention within the booklet group and booklet & seminar group. As for attitude, for the control group, the eta squared value was 0.49 indicated a large size effect. Parents/guardians in the study groups of attitudes towards drowning prevention and water safety showed a significant change after the four weeks. The comparison of the change in the practice of parents/guardians towards drowning prevention and water safety from baseline to four weeks after intervention showed the control group has the mean knowledge decreased significantly at post-test.

**Table 2:** Comparison of knowledge, attitude and practice mean scores in control and intervention groups from baseline to four weeks after intervention

***Variable groups***	***Pre-test (N=719)***		***Post-test (N=386)***		***Mean change***	***t-value***	***P-value***

	***n***	***Mean (±SD)***	***n***	***Mean (±SD)***			
Knowledge							
Control	384	20.22 (3.58)	197	22.11 (3.78)	1.89	4.808	0.000^*^
Booklet group	174	22.22 (2.74)	103	27.72 (2.99)	5.51	12.78	0.000^*^
Booklet & seminar group	161	21.73 (3.45)	86	26.51 (1.94)	4.78	10.86	0.000^*^
Attitude							
Control	384	82.08 (6.92)	197	95.89 (16.60)	13.82	10.79	0.000^*^
Booklet group	174	82.49 (6.05)	103	106.15(15.08)	23.66	14.49	0.000^*^
Booklet & seminar group	161	81.90 (6.58)	86	104.50(10.30)	22.61	14.01	0.000^*^
Practice							
Control	384	112.72 (11.94)	197	104.18 (17.80)	−8.54	−5.49	0.000^*^
Booklet group	174	112.00 (12.85)	103	112.68 (12.59)	0.68	0.41	0.682
Booklet & seminar group	161	109.69 (15.08)	86	104.56 (10.30)	−5.13	−2.36	0.021^*^

The eta squared was 0.13 indicated the small effect size. In overall, after an intervention, the practice scores decreased in the control and also the booklet & seminar group significantly but with a very small size effect while in the booklet group the practice score was not significantly different from before and after the intervention.

### Effect of the intervention

#### Between and within-group comparison of knowledge, attitude and practice after intervention

Repeated measures ANOVA test was applied to indicate if the differences in change of knowledge, attitudes and practice scores between pre-test and post-test were significantly different in the control and intervention groups. The Mauchly’s test resulted in a violation of the assumption of sphericity; thus the Greenhouse-Geisesser corrected estimated were used in interpreted results.

The results determined that mean scores knowledge has a significant interaction between group and time point (F (2, 383) = 22.82, *P* <0.001), with large effect size (ƞ2 =0.106). The overall main effect for time was significant with F (1, 383) = 237.89, *P*<0.001 and large effect size (ƞ2 =0.38). The overall group effect was significant with F (2, 383) = 123.95, *P*<0.001 and large effect size (ƞ2 =0.39).

**Fig. 3:**
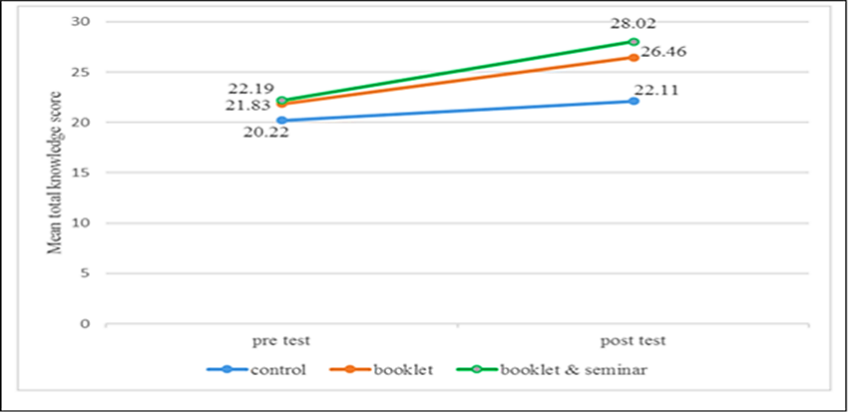
Changes in mean total knowledge score between and within the study group

**Fig. 4:**
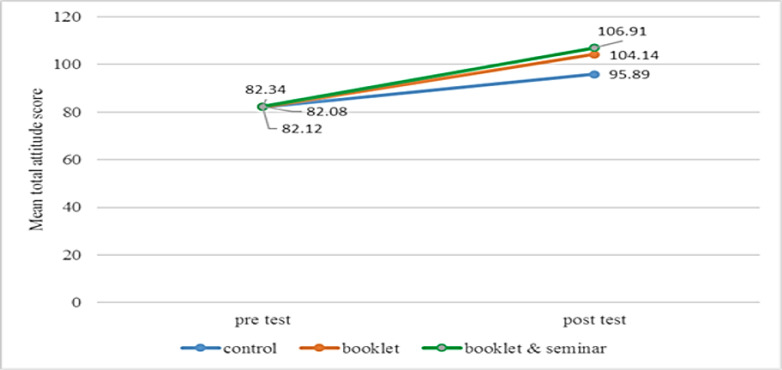
Changes in mean total attitude score between and within the study group

Fig. 3 shows a comparison of mean score changes in knowledge between study groups across the study. The mean total score of attitudes differed statistically significantly between group and time points (F (2, 383) = 15.23, *P* < 0.0001) with large size effect of 0.74. The overall significant main effect for time was F (1, 383) = 480.42, *P* < 0.0001) with large effect size (ƞ2 =0.106). The main effect of the group also showed a significant difference with F (2, 383) = 16.79, *P*<0.001 with small size effect (ƞ2 =0.08). A comparison of mean score changes of attitudes between the study group and time were presented in Fig. 4.

**Fig. 5:**
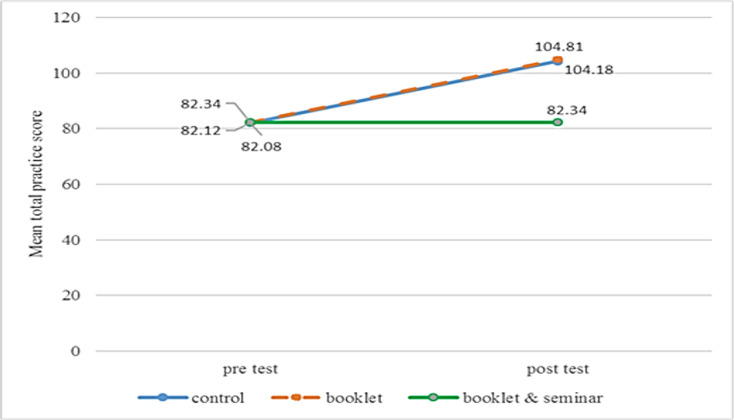
Changes in mean total practice score between and within the study group

The results for practice shows a significant interaction between group and time with F (2, 383) = 10.98, *P*<0.001 with medium size effect (0.05). There was an overall significant main effect for time, F (1, 383) = 828.44, *P*<0.001 and large effect size of 0.68. Similarly, the main effect for the group also was found significant with F (2, 383) = 11.95, *P*<0.001 and a medium-size effect of 0.06. The comparison of mean score changes in practice towards drowning prevention and water safety between the study group and time were illustrated in Fig. 5.

There were a significant interaction between time and group was found for knowledge, attitude and practice that suggests at different time intervals groups performed statistically different and the effect of time within group and effect of intervention were significantly correlated to changes in knowledge, attitude and practice towards drowning prevention and water safety.

## Discussion

Health educational Be SAFE booklet, an integrated parent-based intervention was piloted among parents/guardians of primary school children in Hulu Langat district, Selangor. This is because the levels of parents/guardian’s knowledge, attitude and practice (KAP) towards drowning prevention and water safety have been previously neither analyzed nor reported in this country. Note that this study is the first study that attempted to estimate parents/guardian’s behavioral changes through health education booklet intervention in Malaysia. Findings of this current study support the effectiveness of health educational Be SAFE booklet in improving knowledge on drowning prevention and water safety among parents/guardians of primary school children in Hulu Langat district. Booklet group showed significant improvement knowledge on drowning prevention and water safety with a positive change in the mean score from 22.22 to 27.72 and the change was 24.75% increase in level of knowledge over the four weeks after intervention. The booklet & seminar group also showed a similar result with a significant increase in knowledge means score with 21.73 to 26.51 and the change was 21.9% after one-month intervention. The significant increase in knowledge level seen in both intervention groups, one-month post intervention compared to baseline, but not observed in the control group. These could be attributed to the availability of information obtained from the health educational materials and the seminar on drowning prevention and water safety which was conducted for the intervention group. Similarly, this current study in line with a study in Brazil on short period educational intervention that had presented a significant increase in awareness with related to prevention of childhood injury such as fall, drowning and intoxication (12). Other studies also demonstrated the effectiveness of the educational intervention on change of knowledge on drowning prevention and water safety (6,11–13). In a nutshell, these findings reaffirmed that it is important for parents or guardians to be knowledgeable about the inherent risk of injury in order to expand learning and risk identification regarding accident prevention in childhood since ignorance predispose them to the occurrence of injuries such as drowning among their children.

Attitudes level also demonstrated an increase significantly greater in the intervention group than the control group. Over the four weeks, the mean score of the attitudes of intervention participant’s booklet group significantly increased from 82.49 to 106.15. In intervention participant’s booklet & seminar group also increase significantly with 81.90 to 104.50. Consistently, findings from a study in New Zealand have also shown after education intervention, inappropriate attitude towards drowning prevention changed and decreased (11,12).

In spite of positive changes in knowledge and attitude, this study revealed the effect of the intervention on the practice of drowning prevention and water safety did not present as expected. Although drowning prevention and water safety practice mean score in the booklet group showed some changes after the intervention, from 112.00 to 112.68, it was not significant. As for the booklet & seminar group, the mean score for practice on drowning prevention and water safety were measured low after the intervention compared to the baseline, from 109.69 to 104.56. The control group also showed a slightly lower mean score of practice from 112.72 to 104.18.

Respondent’s practices on drowning prevention and water safety as they relate to issues that cause the child drowning did not change significantly after the intervention. The possible explanation for this might be attributed to a few barriers to change among parents/guardians with regards to drowning prevention and water safety. There are many barriers to change as suggested in the guideline of “How to Change Practice” such as awareness and knowledge, motivation, acceptance and beliefs, skill, practicalities and barrier that beyond our control (14). With related to practice on drowning prevention and water safety, the possible explanation of barriers that might present was the skill. For example, skill in cardiopulmonary resuscitation (CPR) as an elements that is vital for parents/guardians as they are commonly the first responder at the scene of an emergency, yet need time to learn and practice as presented to be limited understanding of child CPR protocols and most parents/guardians felt anxious to perform CPR (11,12).

The other reason may due to motivation that related to priorities and commitment. Parents/guardians might want to provide their children with swimming lesson and water safety skill, but due to high commitment as the swimming class involved some cost, parents/guardians have to put the priorities on the things that are more important and urgent to them. Acceptance and beliefs were also might be the barrier for parents/guardians to practice drowning prevention and water safety for their children. For example, the important use of life-jackets for water-related activity in this country is still not a culture. Mostly as we can see in public beaches or recreational water places such as waterfall and waterpark, there is no life-jacket provided but along the beaches or near the waterpark, the several shops selling all kind of size of water ring. Although the interventions used may not differ with other intervention (life jackets and water awareness), researcher aware that this study reinforces the need for educational messages to be designed keeping cultural beliefs and practices (15).

Another barriers that might exist were awareness and knowledge, even though after the intervention the knowledge and awareness on drowning prevention were higher, but knowledge at the time not necessarily ensure their practices until and after the events happened (16).

Therefore, to overcome this barrier, an improvement of the educational materials and conferences, workshops, training courses and lectures may be one of the solutions to ensure parents/guardians able to practice of drowning prevention and water safety (14,16).

## Conclusion

Parents/guardian’s knowledge regarding the prevention of child drowning and water safety, and the perception of drowning risk improved by health educational intervention (Be SAFE booklet). The scale-up of this intervention with the similar result is quite feasible considering the simple, written, low-cost and reachable of the health educational Be SAFE booklet to all layer of the community including people in the rural area in this country. On top of that, integration with the local authority, Fire and Rescue Department, Safe Kids Malaysia and Life-Saving Society Malaysia (LSSM) in the Be SAFE seminar enhanced the effective preventive measure on drowning.
